# Implication of the Whitefly *Bemisia tabaci* Cyclophilin B Protein in the Transmission of *Tomato yellow leaf curl virus*

**DOI:** 10.3389/fpls.2016.01702

**Published:** 2016-11-15

**Authors:** Surapathrudu Kanakala, Murad Ghanim

**Affiliations:** Department of Entomology, The Volcani CenterRishon LeZion, Israel

**Keywords:** TYLCV, *Bemisia tabaci*, Cyclophilin B, immunostaining, virus transmission

## Abstract

*Tomato yellow leaf curl virus* (TYLCV) is a single-stranded (ssDNA) begomoviruses that causes severe damage to tomato and several other crops worldwide. TYLCV is exclusively transmitted by the sweetpotato whitefly, *Bemisia tabaci* in a persistent circulative and propagative manner. Previous studies have shown that the transmission, retention, and circulation of TYLCV in its vector involves interaction with insect and endosymbiont proteins, which aid in the transmission of the virus, or have a protective role in response to the presence of the virus in the insect body. However, only a low number of such proteins have been identified. Here, the role of *B. tabaci* Cyclophilin B (CypB) in the transmission of TYLCV protein was investigated. Cyclophilins are a large family of cellular prolyl isomerases that have many molecular roles including facilitating protein-protein interactions in the cell. One cyclophilin protein has been implicated in aphid-luteovirus interactions. We demonstrate that the expression of *CypB* from *B. tabaci* is altered upon TYLCV acquisition and retention. Further experiments used immunocapture-PCR and co-immunolocalization and demonstrated a specific interaction and colocalization between CypB and TYLCV in the the midgut, eggs, and salivary glands. Membrane feeding of anti-CypB antibodies and TYLCV-infected plants showed a decrease in TYLCV transmission, suggesting a critical role that CypB plays in TYLCV transmission. Further experiments, which used membrane feeding with the CypB inhibitor Cyclosporin A showed decrease in CypB-TYLCV colocalization in the midgut and virus transmission. Altogether, our results indicate that CypB plays an important role in TYLCV transmission by *B. tabaci*.

## Introduction

Begomoviruses are a group of icosahedral single-stranded DNA (ssDNA) viruses exclusively transmitted by the whitefly *Bemisia tabaci* in a persistent, circulative manner. Among the whitefly-transmitted viruses, 90% belong to the genera *Begomovirus* (Jones, 2003), which includes approximately 288 species^1^, and have emerged as the most threatening group of plant viruses globally during the past two decades, as reported from dicotyledonous host-causing diseases of economic importance (Brown and Czosnek, 2002). Of all tomato begomoviruses, *Tomato yellow leaf curl virus* (TYLCV) is the most threatening to tomato production worldwide (Czosnek, 2007). TYLCV is exclusively transmitted by *B. tabaci*, and many parameters for the virus acquisition, transmission, and retention have been extensively documented (Rubinstein and Czosnek, 1997; Brown and Czosnek, 2002). The 29-kDa virus coat protein (CP) is exclusively required for transmission and interaction with *B. tabaci* tissues and proteins (Briddon et al., 1990; Noris et al., 1998). TYLCV virions are acquired with the phloem sap, move along the food canal and foregut to reach the midgut, translocate across the gut epithelia into the hemolymph, then enter the primary salivary glands via endocytosis, from which they are expelled into the host plant with salivary secretions (Ghanim, 2014; Gray et al., 2014).

During this process, TYLCV is hypothesized to interact with insect proteins that influence and facilitate the virus transmission (Ghanim et al., 2001; Ghanim, 2014). Recent studies have investigated the importance of TYLCV CP in virus transmission and demonstrated its interaction with a member of the small heat-shock protein family (BtHSP16), which was identified using a yeast-two hybrid system screen against *Tomato yellow leaf curl Sardinia virus* (TYLCSV) CP (Ohnesorge and Bejarano, 2009). Another study has identified another heat-shock protein, HSP70, which interacts with the TYLCV CP *in vivo* and *in vitro*. Membrane feeding with anti-HSP70 antibodies resulted in an increase in TYLCV transmission. This result suggested that under normal conditions HSP70 restricts virus activity and transmission, thereby protecting the insect from deleterious effects of TYLCV (Gotz et al., 2012). Interactions with these proteins may be necessary for refolding of the virion particle and facilitating their circulation and translocation across membrane barriers especially the midgut-hemolymph and the hemolymph-salivary glands.

The identification of additional whitefly proteins that interact with and mediate virus transmission is critical for understanding viral strategies that aid in their highly successful transmission by whiteflies. The search for such proteins in many cases is based on results described in other circulative virus-vector systems in which other proteins facilitating virus transmission have been identified. One such example was demonstrated in recent studies in which genetic and proteomic analyses suggested that virus-binding aphid proteins belonging to the peptidyl-prolyl isomerases proteins (PPIases or Cyps) were specifically inherited and conserved in different aphid vector genotypes (Tamborindeguy et al., 2013). This suggested that they play a major role in regulating *Cereal yellow dwarf virus*-*RPV* (CYDV-RPV) transmission (Yang et al., 2008). Tamborindeguy et al. (2013) showed that both CypA and B interact with CYDV-RPV, and these interactions may be important but not sufficient to mediate virus transport from the hindgut lumen into the hemocoel.

Like aphids, in whiteflies, expressed *Cyp* genes were detected in EST libraries (Saripalli, 2008), and those were amplified from TYLCV non-viruliferous *B. tabaci* B adults. Members of the PPIase protein family (e.g., cyclophilins, FKBPs, and parvulins) are enzymes found in both eukaryotes and prokaryotes, participate in cell signaling, gene transcription and assist folding and localization of proteins, respectively (Hanes, 2015). More recently, Cyps were shown to facilitate dissociation of the human Papillomavirus Type 16 L1 and L2 capsid proteins from L2/DNA complexes following virus entry (Bienkowska-Haba et al., 2012), while CypA was shown to bind *Tomato bushy stunt tombusvirus* and inhibit tombusvirus replicase assembly (Kovalev and Nagy, 2013). Furthermore, Zhou et al. (2016) showed that *Cyp* genes contribute to the development and virulence of *Beauveria bassiana*, an entomopathogenic fungus. In the case of the whitefly-begomovirus system, interactions with these proteins may be necessary for refolding of virion particle, facilitating interactions with other whitefly proteins, and aiding the virus in crossing membrane barriers in the whitefly. In whiteflies, nothing is known about the function of Cyp proteins and whether they play a role in virus-whitefly interactions. In the current study we used an arsenal of biological and molecular methods to demonstrate that CypB, a member of this protein family plays an important role in *B. tabaci*-TYLCV interactions.

## Materials and Methods

### Maintenance of Whiteflies and Plants

Population of the *B. tabaci* B infected with the secondary symbionts *Rickettsia* and *Hamiltonella*, rearing conditions and establishment of iso-female strains of *Rickettsia*-free (R-) and *Rickettsia*-infected (R+) strains, were conducted as previously described (Brumin et al., 2012). Briefly, the populations were reared on cotton seedlings (*Gossypium hirsutum* L. cv. Acala) maintained inside insect-proof cages and growth rooms under standard conditions of 25 ± 2°C, 60% relative humidity and a 14 h light/10 h dark photoperiod. The purity of the *B. tabaci* B population was confirmed by PCR with Bem 23 primers (**Table 1**). Healthy cotton plants were added once a month, while older plants were cut 2 days before, to make sure all the whiteflies move to the newly added plants. Both R- and R+ populations were handled in alternate days to avoid cross contamination. Unless otherwise indicated, all experiments in this work were conducted with the R+ strain.

**Table 1:** Primers used in this study.

Gene/Probe	Name	Primer sequence (5′→3′)	Expected product size (bp)	Reference
CypB	CypBF	ATGAAGAACCCGAAAGTTCA	615	This study
	CypBR	TTATTCGGTGGCATCAGCTT		
CypBs	CypBsF	ATGAAGAACCCGAAAGTTCA	216	This study
	CypBsR	GAAATTCTCGACAGTCTTCG		
CypDs	CypDsF	ATGGAGCTCCGCAATGATGT	155	This study
	CypDsR	GTGCCGTTGTGGTTTGTGAA		
CypGs	CypGsF	AGATGTACCGCAGCCCAAAT	188	This study
	CypGsR	GGGTCCGATACCTCAGGACT		
*Bemisia tabaci* β-Actin	Fβ-Actin	TCTTCCAGCCATCCTTCTTG	81	Sinisterra et al., 2005
	Rβ-Actin	CGGTGATTTCCTTCTGCATT		
*B. tabaci* B	Bem 23F	CGGAGCTTGCGCCTTAGTC	200	De Barro et al., 2003
	Bem 23R	CGGCTTTATCATAGCTCTCGT		
TYLCV CP	V61	ATACTTGGACACCTAATGGC	412	Ghanim et al., 1998
	C473	AGTCACGGGCCCTTACA		

Tomato (*Solanum lycopersicum* cv. Avigail) and cotton plants were grown in potting mix in 1.5-L pots under artificial light and maintained inside insect-proof cages in the greenhouse under controlled conditions as detailed above.

### TYLCV Source and Insect-Mediated Transmission

Partial tandem repeat (PTR) construct of an Israeli isolate of TYLCV DNA A (Genbank Accession number X15656) described previously in Navot et al. (1991) was used. PTR construct was transformed to the *Agrobacterium tumefaciens* strain C58 by direct transformation. A 2-day-old *A. tumefaciens* culture carrying the construct was plated on LB broth with rifampicin (30 mg/ml) and kanamycin (50 mg/ml), and incubated at 28°C for 24 h (180 rpm), the *Agrobacterium* cells were collected by centrifugation for 10 min at 3,000 rpm and resuspended to an OD600 of 0.6–0.9 in suspension solution (MS medium supplemented with 10 mM MES and 200 mM acetosyringone) and incubated at room temperature for 2 h before agroinfiltration. The tomato plants were observed for leaf curl symptoms and subjected to PCR after 3 weeks using TYLCV-specific primers V1 and C473 (Ghanim et al., 1998). PCR positive plants were maintained under insect-free cages for further studies. Symptomatic leaves were used for immunocapture-PCR (IC-PCR) and virus acquisition and transmission experiments.

### DNA and RNA Extraction from *B. tabaci*

Genomic DNA was isolated from single *B. tabaci* B adults using the CTAB (cetyltrimethylammonium bromide) method (Shahjahan et al., 1995). Briefly, single whitefly was homogenized in 30 μl of extraction buffer (10 mM Tris-HCl, 1.4 M NaCl, 2 mM EDTA, 2% CTAB, and 0.2% β-mercaptoethanol) and incubated at 65°C for 15 min, followed by incubation at 95°C for 10 min. Samples were centrifuged for 55 min at 16,300 × *g*, and an equal volume of phenol – chloroform – isoamyl alcohol (25:24:1) was added. The aqueous phase was transferred to clean tube, equal volumes of chloroform were added, followed by another centrifugation. The upper phase was collected, and nucleic acids were precipitated using 0.2 volumes of 5 M sodium chloride (NaCl) and one volume of isopropanol. The samples were incubated at 4°C overnight for DNA precipitation. The samples were then centrifuged at 14,000 × *g* for 30 min at 4°C. The pellet was washed with 70% ice-cold ethanol, air-dried and dissolved in 40 μl of double-distilled water.

For total RNA extractions, each replicate out of the three, which consisted about forty 6- to 7-day-old adults, was homogenized in 300 μl of Tri-reagent (Sigma), and 1 μl of polyacrylamide carrier (GenElute LPA; Sigma) was added and the tube vortexed for 10 s and incubated for 5 min at room temperature. Sixty microliters of chloroform were added, followed by vigorous mixing and centrifugation at 12,000 × *g* for 10 min at 4°C. The aqueous phase was transferred to a clean tube, gently mixed with 0.7 volumes of ice-cold isopropanol, and incubated overnight at -20°C for RNA precipitation. The samples were centrifuged at 14,000 × *g* for 30 min at 4°C. The supernatant was removed and pellet was washed with 75 % ice-cold ethanol. The air-dried pellet was dissolved in 40 μl of double-distilled water. DNA and RNA purity and yield were analyzed using a NanoDrop 1000 spectrophotometer (Thermo Fisher Scientific).

### Plant DNA Extraction

Total DNA was extracted from 100 mg leaves of tomato plants showing typical leaf curl symptoms. The young leaf tissues were ground in liquid nitrogen using pestle and mortar and the homogenate was transferred to modified extraction buffer (100 mM Tris-HCl, pH 8.0, 1.4 M NaCl; 20 mM EDTA, 2% CTAB, and 2% β-mercaptoethanol). The homogenized sap was transferred into microfuge tubes and incubated at 65°C for >30 min and mixed by inversion 3–5 times. Equal volume of chloroform:isoamyl alcohol (24:1) was added into each tube and tubes were mixed gently by inversion for 10 min followed by centrifugation at 13,000 rpm for 10 min at 4°C. The aqueous phase was transferred to a sterile microfuge tube. To the aqueous phase, 0.8 volume of isopropanol was added and mixed gently to precipitate the nucleic acids. The DNA was separated by centrifugation at 13,000 rpm for 10 min at 4°C, followed by washes with 80 and 70% ethanol, separately. The pellet was air-dried, and dissolved in 50 μl of double-distilled H_2_O. The DNA was further purified by RNase A treatment and stored at -20°C for further use.

### Preparation of cDNA from RNA and Whitefly Midguts

RNA (100 ng) was used as a template for cDNA synthesis in 25-μl reaction mixtures by using Verso cDNA kit (Thermo scientific, Fermentas). Each of the three biological replicates used RNAs from 10 dissected midguts for cDNA synthesis. Dissected midguts were washed using PBST (1X PBS with 0.05% [vol/vol] Tween 20) twice and dissolved in 20 μl of RNase*-*free double-distilled water. The samples were then incubated at 95°C for 10 min to disrupt the cells and ∼4 μl of the cells (not purified RNA) were used for cDNA synthesis. For midgut cDNA synthesis, Maxima kit with dsDNase (Thermo Scientific) was used.

### Gene Amplification, Sequence, and Phylogenetic Analysis

PCR primers for amplifications were designed using sequences obtained from *B. tabaci* transcriptome sequence data generated through several projects performed in our lab, and sequences available in GenBank, and were confirmed with the new assembled whitefly genome (Drs. Zhangjun Fei and Wenbo Chen, personal communication). PCR amplifications were performed using *CypB, CypB*s, *CypD*s, and *CypG*s forward and reverse primers (**Table 1**) with predicted sizes of 615, 216, 155, and 188 bp, respectively. The PCR mixture consisted of 50 ng of cDNA, 1 mM of the forward and reverse primer, 1.5 mM MgCl_2_, 0.25 mM of each dNTP, 2 units of Taq DNA polymerase (Hylabs, Rehovot, Israel), and the final volume was made up to 20 μl with sterile distilled water. PCR cycling conditions were as follows: 94°C for 3 min, 30 cycles of 94°C for 30 s, 54°C for 30 s, 72°C for 30 s, and final extension at 72°C for 5 min. The PCR product was resolved in 1% agarose gel, and 1-kb DNA ladder (Thermo Scientific) was used as a molecular weight marker. PCR products were extracted from the gel and sent for purification and sequencing (Hylabs, Rehovot, Israel).

### Sequence and Phylogenetic Analysis

*Bemisia tabaci* B *Cyp* nucleotide and amino acid sequences were initially analyzed by using the BLASTN and BLASTP algorithm at NCBI website^2^ and the Expert Protein Analysis System^3^. The 3D structure of CypB was predicted using SWISSMODEL^4^. Sequences were aligned with closely related arthropod Cyp sequences obtained from the Genbank sequences database using Bioedit program^5^. The predicted amino acid sequence of the protein with CypBs from *Melanoplus sanguinipes, Zootermopsis nevadensis, Cyphomyrmex costatus*, and *Tribolium castaneum* showed identity of 69, 68, 67, and 66%, respectively. A phylogenetic tree was generated using a neighbor-joining algorithm bootstrapped with 1000 replicates in MEGA6 (Tamura et al., 2013) to evaluate the statistical robustness of the internal tree branches.

### qRT-PCR Analysis

To measure the expression levels of the *CypB, CypD*, and *CypG* genes, a quantitative RT-PCR (qRT-PCR) approach was used. The primer pairs used for the amplifications are listed in **Table 1**. Amplifications were performed using Absolute Blue qPCR SYBR green Rox mix (Thermo Scientific) and 5 pmol of each primer. To ensure the validity of the data, the expression of each gene was tested in triplicate in each of three biologically independent experiments. The cycling conditions were as follows: 15 min of activation at 95°C and 40 cycles of 15 s at 95°C, 30 s at 58°C (actin), 54°C (*CypB, CypD*, and *CypG*), and 30 s at 72°C. The channel source was 470 nm, with a detector at 510 nm. A Rotor-Gene 6000 machine (Corbett Robotics Pty. Ltd., Brisbane, QLD, Australia) and the accompanying software were used for qPCR data normalization and quantification. Average expression of actin cDNA, which was not regulated after virus acquisition experiments, was used as a reference as stated above, and the quantification was done using the relative expression method. For each run, standards were loaded onto the same plate to obtain the appropriate standard curve.

### Immunocapture-PCR

Interaction between CypB and TYLCV CP was tested by IC-PCR assay. The wells of microtiter plates were coated with coating buffer (0.05 M sodium carbonate pH 9.5) including a polyclonal antibody against cyclophilin (Cyp) IgG1g/ml (Sigma–Aldrich catalog # SAB4200201) whose specificity was confirmed using western blot analysis in which an expected band of ∼20 KDa was obtained (data not shown). Anti-TYLCV CP antibody (a gift from Prof. Henryk Czosnek) was also used. The plated were coated with both antibodies for 4 h at room temperature. About 30 viruliferous whitefly adults or 100 mg leaf tissue were crushed in one volume of extraction buffer, 50 mM Tris–HCl (pH 8.0), 5 mM EDTA, 2% polyvinylpyrrolidone, and 0.05% Tween 20. The extract was clarified at 13,000 × *g* for 15 min at 4°C. The clarified extract was treated with DNase I (1 U/l) for 30 min at 37°C to remove the viral replicative DNA to ensure that only encapsidated ssDNA served as the template (Kanakala et al., 2013). The plates were washed with 1X PBS three times to remove unbound antibody, and 200 μl aliquots of DNase treated plant and whitefly extract were added into the wells in three replicates. The plate was incubated overnight at 4°C to allow maximum capturing of the particles. The overnight-incubated plates were washed with 1X PBS to remove all unbound material. The bound particles were released by adding 50 μl/well of extraction buffer with 1% (v/v) Triton X-100 and the suspension was stored at 4°C until further use. PCR amplification of the 5 μl of viral DNA from the virions bound to the CypB protein, which was itself bound to the antibody-coated tubes, was performed with the TYLCV-specific primers V1 and C473 (Ghanim et al., 1998).

### Immunostaining of TYLCV and CypB in *B. tabaci* B Midguts, Salivary Glands, and Eggs

Whitefly midguts, salivary glands and eggs were dissected on glass microscopic slides, and fixed with 4% paraformaldehyde in 1X PBS buffer (10 mM Tris HCl, 150 mM sodium chloride, pH 7.5) for 30 min at room temperature, followed by 200 μl of 0.1% triton-X100 for 0.5–1 h at room temperature. After washing with PBST (1X PBS with 0.05% [vol/vol] Tween 20) for three times, the specimens were blocked with 200 μl of blocking buffer (PBST+ 1% Bovine Serum Albumin, BSA) for 1–2 h at room temperature. Then TYLCV CP (Gorovits et al., 2013) primary antibody diluted in 1:100 in blocking buffer was incubated with the samples at 4°C overnight. After washing with PBST, samples were incubated for 1–2 h at 25°C with a Cy3-conjugated anti-rabbit secondary antibody (Jackson ImmunoResearch, USA) diluted to 1:200. The samples were washed three times and blocked for 2 h. Then anti-CypB primary antibody (Sigma–Aldrich SAB4200201) diluted in 1:100 in blocking buffer was incubated with the samples overnight at 4°C. After washing with PBST, samples were incubated for 1–2 h at 25°C with a Cy2-conjugated anti-rabbit secondary antibody (Jackson ImmunoResearch, USA) diluted 1:200. After washing three times with PBST, the nuclei were stained with DAPI in PBST (Thermo Scientific DAPI, Pierce Protein Research Product), at 1 μg/ml for 20 min at 25°C, covered with a cover-slip, sealed with nail polish and viewed under aIX81 Olympus FluoView500 confocal laser-scanning microscope; TYLCV CP and CypB were detected as red and green fluorescent signals, respectively. For each treatment, 15–20 whitefly midguts, salivary glands, and eggs were viewed. Controls consisted of performing the same experiments but not including first antibodies, and also switching the order of using the primary antibodies to ensure specificity in binding, and to ensure that both secondary antibodies saturate the primary ones in each step, because both primary antibodies were raised in rabbit. Another control consisted performing the protocol without using the anti-CypB antibody as detailed in the results.

### Transmission of TYLCV after Feeding on CypB Antibodies and Cyclosporin A

To assess the implication of CypB in the transmission of TYLCV, *B. tabaci* adults, 1 week after emergence, were fed on antibodies against CypB (2 μg/μl) or the Cyp inhibitor Cyclosporin A (CsA) (2 μg/μl; Sigma–Aldrich C1832). The antibody or the inhibitor were mixed with 25% sucrose solution and confined between two layers of stretched parafilm M (Bemis, Neenah, WI, USA) on a glass scintillation vial in which the adult whiteflies were caged for feeding on this solution for 24 h. The insects were then transferred to TYLCV-infected tomato plants for a 48-h acquisition access period and subsequently single whiteflies were transferred to tomato plants in their three-leaf stage for 7 days of inoculation access period. The single whiteflies were confined to the plants in leaf-clip cages. Whiteflies fed on 25% sucrose solution for 24 h and TYLCV-infected plants for 48 h served as a control. Tomato plants were grown in a potting mix in 1.5-L pots under artificial light and maintained inside insect-free greenhouse under controlled temperature as detailed above. The whiteflies that were incubated with the plants were tested for TYLCV acquisition. The plants were monitored for the development of disease symptoms after 28 days post inoculation. DNA was extracted from symptomatic and non-symptomatic tomato plants and subjected to PCR for detecting TYLCV with specific CP primers V61 and V473 (Ghanim et al., 1998). The experiments were triplicated with 20 plants for each replicate.

### Statistical Analyses

The significance of the differences between means in all comparisons performed on data from the qRT-PCR and virus transmission experiments was determined by replicating the experiments at least three times and using the Tukey–Kramer honest significance difference (HSD) test (α = 0.05). JMP7 (SAS institute) was used for all statistical analysis. In all of the figures, asterisks indicate significant differences and the significance *p <* 0.05.

## Results

### Sequence Analysis of *Cyp* Genes of *B. tabaci* B

**FIGURE 1:**
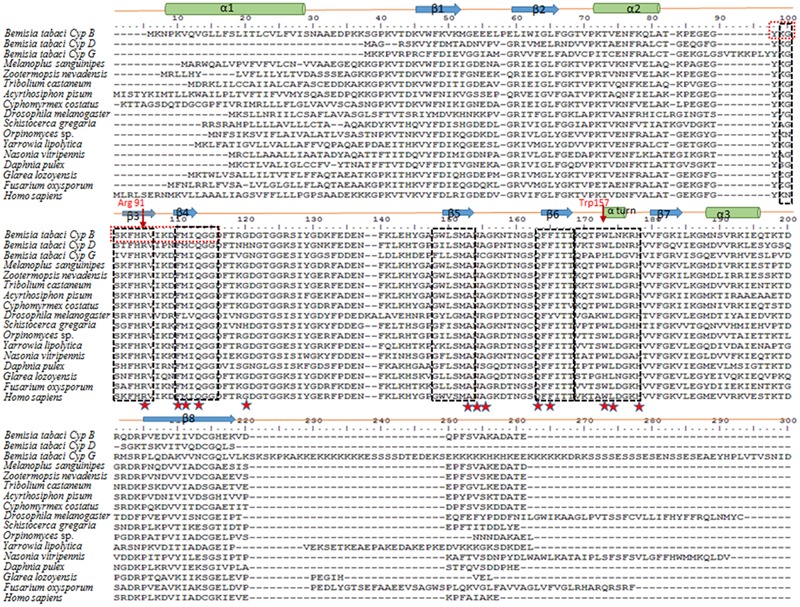
**Multiple alignments of *Bemisia tabaci* CypB, CypD, and CypG amino acid sequences with other arthropod, fungal, and human CypB amino acids sequences. ‘β 1–8’ indicates β-strand; ‘α 1–3’ indicates α-helix; The CypB inhibitor cyclosporin A (CsA)-binding domains (four β-strands) are indicated on the top of sequences and are boxed.** The 13 well-conserved residues that constitute the CsA-binding site are marked with star symbol. The signature of peptidyl-prolyl-*cis*-*trans* isomerase (YKGSKFHRVIKDFMIQGG) is represented in red colour box. Accession numbers are: *Melanoplus sanguinipes* ALX00032, *Zootermopsis nevadensis* KDR13508, *Tribolium castaneum* XP 971028, *Acyrthosiphon pisum* NP 001156707, *Cyphomyrmex costatus* KYN04146, *Drosophila melanogaster* NP 476656, *Schistocerca gregaria* AEV89763, *Orpinomyces sp.* Q01490, *Yarrowia lipolytica* XP 504296, *Nasonia vitripennis* XP 001602615, *Daphnia pulex* EFX68543, *Glarea lozoyensis* EHK99826, *Fusarium oxysporum* EXL62949, *Homo sapiens* P23284.

*Cyp* EST sequences were obtained from several deep-sequencing projects with *B. tabaci* generated in our lab with the Illumina technology (not published), and were confirmed using the Nr or Swiss-Prot databases, and were finally confirmed with gene sequences from the whitefly genome project. Initially, *CypB* was amplified from whiteflies using corresponding primer pairs (**Table 1**), which yielded a single band of 615 bp. The complete gene sequence of *CypB, CypD*, and *CypG* cDNAs were recovered from the available sequences obtained in our sequencing projects. The complete sequence of *CypB* encodes a polypeptide of 216 amino acids with a predicted molecular weight of 24.15 kDa and an isoelectric point of 8.076. Alignment of the deduced amino acid sequences of the three *Cyp* genes revealed strong identities with those of other *CypB*s genes of other arthropods obtained from Genebank (**Figure 1**). The nucleotide and deduced amino acid sequence of the *B. tabaci CypB, CypD*, and *CypG* have been deposited in GenBank under the accession number**s** KX268377, KX268378, and KX268379, respectively.

**FIGURE 2:**
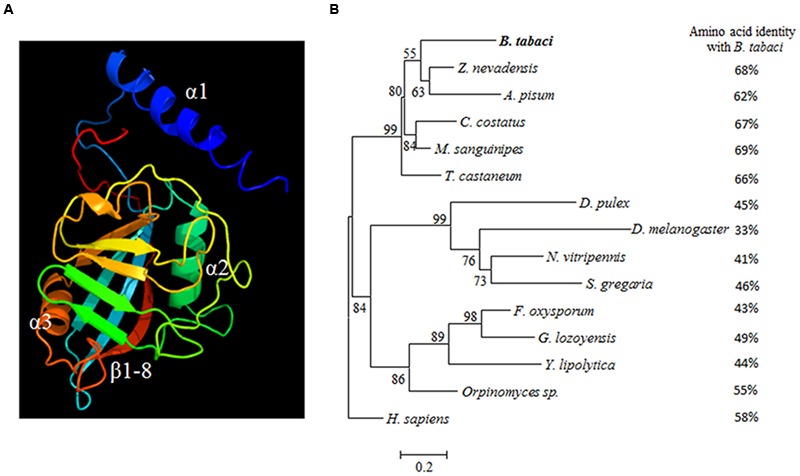
**(A)** The 3-D structure of *B. tabaci*-CypB. The β-strands and α-helices are shown. **(B)** Phylogenetic tree of *B. tabaci*-CypB and other arthropods, fungal species and *H. sapiens*, were constructed using MEGA 6 with maximum likelihood method. Numbers next to the branches indicated bootstrap value of each internal branch in the phylogenetic tree nodes from 1000 replicates. Cyclophilin B sequences include *Zootermopsis nevadensis (*KDR13508); *Acyrthosiphon pisum* (NP 001156707); *Cyphomyrmex costatus* (KYN04146*); Melanoplus sanguinipes (*ALX00032); *Tribolium castaneum* (XP 971028); *Daphnia pulex* (EFX68543); *Drosophila melanogaster* (NP 476656); *Nasonia vitripennis* (XP 001602615); *Schistocerca gregaria* (AEV89763); *Fusarium oxysporum* (EXL62949); *Glarea lozoyensis* (EHK99826); *Yarrowia lipolytica* (XP 504296); *Orpinomyces* sp. (Q01490), and *Homo sapiens* (P23284). Amino acid sequence identity with other arthropods and fungal species and *H. sapiens* are given for easy comparison.

Further analysis on the Cyp protein sequence from *B. tabaci*, and amino acid sequences from five arthropods and four fungal species revealed the presence of a conserved domain of the protein. This single domain of Cyp PPIase is located between the amino acids 84–101 (YKGSKFHRVIKDFMIQGG) in CypB and was found to be highly conserved in *B. tabaci* and few other arthropods as shown in the deduced polypeptide of *B. tabaci*-CypB protein (**Figure 1**). The conserved amino acids residues (Y84, R91, and F96) are necessary for the function of the CypB PPIase activity. The 13-highly conserved CsA-binding residues (named R91, F96, M97, Q99, G108, A137, N138, A139, Q147, F149, W157, L158, and H162) were also found in *B. tabaci* CypB. In *B. tabaci* CypB, fold architecture resembled structures from other organisms from higher animals (*Bos taurus*) to unicellular parasites (plasmodium), and consisted of eight antiparallel β sheets and three α-helices that pack against the sheets. Among them four β-strands (K88-I93, F96-I98, W133-A137, F148-T151) and a loop (Q154-H162) that forms CsA-binding pocket (**Figure 1**). In addition, a short α-helical turn containing the active site residue Trp157 found in the β6–β7 loop region is also present (**Figure 2A**). To further validate the molecular evolution on the multiple alignments of CypBs, a phylogenetic tree was constructed based on the amino acid sequences of the protein sequences using maximum likelihood method (**Figure 2B**). According to the phylogenetic analysis, *B. tabaci* firstly clustered with CypB of *Z. nevadensis and Acyrthosiphon pisum* and then formed a sister group with CypBs from other arthropods. Subsequently, they clustered with CypBs from fungal species.

### Expression Analysis of *Cyp* Genes in *B. tabaci* B

**FIGURE 3:**
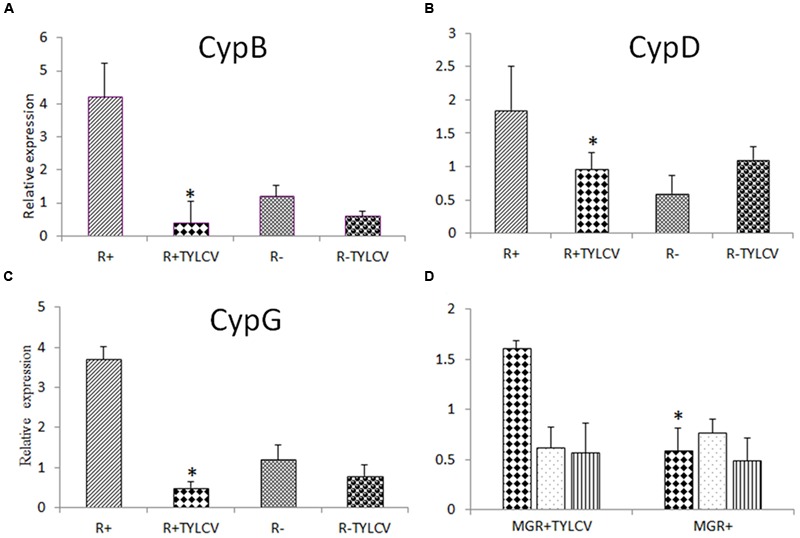
**Relative expression of cyclophilins in viruliferous and non-viruliferous whiteflies (NVBt) measured using quantitative RT-PCR (qRT-PCR), compared to the expression levels of the housekeeping gene β-actin gene. (A–C)** Show relative expression of CypB, CypD, and CypG in viruliferous and NVBt, respectively, in *Rickettsia*-infected (R+) and uninfected (R-) whiteflies. Whiteflies were given acquisition access period for 10 h on *Tomato yellow leaf curl virus* (TYLCV)-infected plants, or healthy plants as control. **(D)** Relative expression of CypB (triangle columns), CypD (dotted columns), and CypG (vertical lines columns) in *B. tabaci* dissected midguts from viruliferous (MGR+TYLCV) and non-viruliferous (MGR+) *Rickettsia-*infected whitefly B adults after a 10 h acquisition access period on TYLCV-infected and healthy tomato (HT), respectively. Data shown are the means ± standard errors of the means of data from three independent experiments. Asterisk indicate statistically significant differences with *P* < 0.05 between viruliferous and non-viruliferous insects.

A previous report has suggested that cyclophilins play a role in aphid-luteovirus interactions. It was thus decided to measure the expression of *Cyp* genes from *B. tabaci* B after TYLCV acquisition. The expression of all three *Cyp* genes from *B. tabaci, CypB, CypD*, and *CypG*, was measured using a quantitative realtime PCR approach, after TYLCV acquisition. All three genes were down regulated after virus acquisition in adults infected with the secondary symbiont *Rickettsia* (R+) (**Figures 3A–C**). However, the expression of the three genes was different in adults lacking the secondary endosymbiont *Rickettsia* (R-) (**Figures 3A–C**). The expression of the three *Cyp* genes was measured in dissected midguts from viruliferous and non-viruliferous R+ adults. Only *CypB* showed significantly higher expression in viruliferous midguts compared to non-viruliferous ones. Based on this result it was decided to further investigate the role of this specific protein in *B. tabaci-*TYLCV interactions (**Figure 3D**).

### Interaction between TYLCV CP and CypB

**FIGURE 4:**
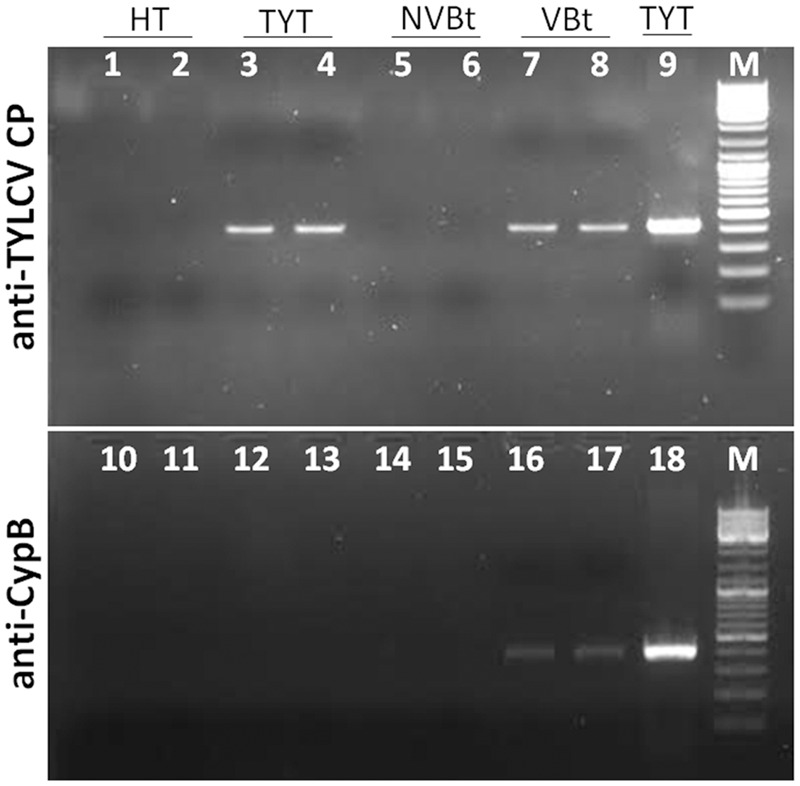
**Immunocapture-PCR assay to detect CypB-TYLCV CP interaction in viruliferous whiteflies (VBt).** Lanes 1–8 and 10–17 represents microtiter plates coated with an anti-TYLCV CP and anti-CypB specific antibodies, respectively, and a PCR was performed on the caught virus-CypB complexes. Lanes 1, 2, 10, and 11 are leaf extract from HT, lanes 3, 4, 12, and 13 are leaf extract from TYLCV-infected tomato (TYT). Lanes 5, 6, 14, and 15 are extracts of NVBt, and lanes 7, 8, 16, and 17 are extracts of VBt. Lane 9 and 18 are genomic extracted from TYT (PCR positive control). TYLCV V61-C473 (**Table 1**) primers were used to amplify the virus bind to the anti-TYLCV CP and anti-CypB antibody.

**FIGURE 5:**
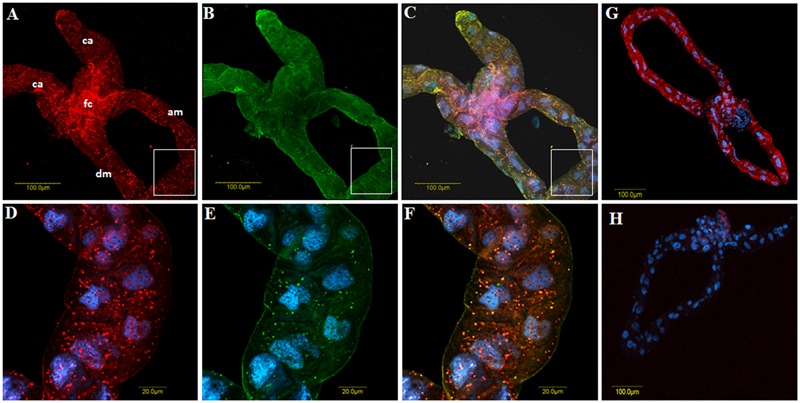
**Co-immunolocalization of TYLCV with first antibody against the virus CP and detected with secondary antibody conjugated to Cy3 (A**, red), and CypB reacted with polyclonal anti-CypB first antibody and detected with secondary antibody conjugated to Cy2 anti-rabbit secondary antibody (**B**, green) in *B. tabaci* midguts dissected from viruliferous adults. **(C)** Shows the overlay of **(A** and **B)** and the yellow spots show the colocalization. **(D–F)** Are zoom in of the portions shown in the insets that appear in **(A–C)**, respectively. **(G)** Is a control gut in which the whole co-immunolocalization procedure was performed without adding the anti-CypB primary antibody, and **(H)** is a control gut in which only secondary antibodies were used. ca, cecae; fc, filter chamber; am, ascending midgut; dm, descending midgut.

**FIGURE 6:**
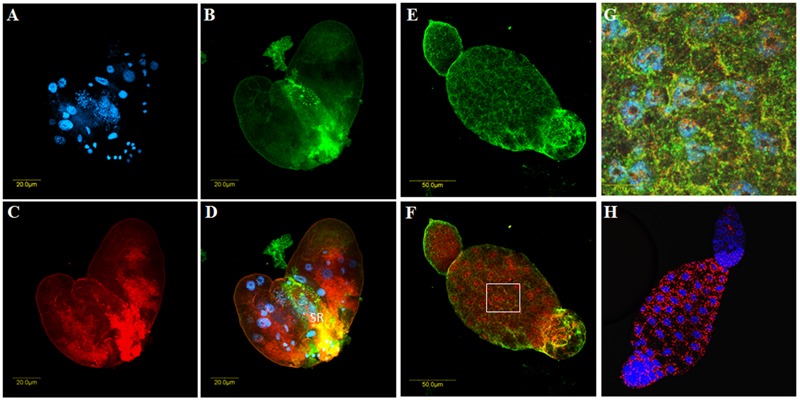
**Co-immunolocalization of TYLCV with first antibody against TYLCV CP of the virus, and detected with a secondary antibody conjugated to Cy3 (red), and first antibody against CypB detected with a secondary antibody conjugated to Cy2 anti-rabbit secondary antibody (green) in *B. tabaci* primary salivary glands (A–D)** and in oocytes **(E–H)** dissected from viruliferous adults. **(A)** Shows one gland stained with DAPI for nuclei, **(B)** shows CypB immunolocalization, **(C)** shows TYLCV immunolocalization, and **(D)** is overlay of **(A–C)** showing the co-immunolocalization of CypB and TYLCV in the yellow portions near the secretory region of the primary salivary gland (SR). **(E)** shows one oocyte in which CypB is immunolocalized, **(F)** shows co-immunolocalization of TYLCV with CypB in the same oocyte, **(G)** is zoom in of the inset in **(F)** and **(H)** is a control oocyte in which the whole co-immunolocalization procedure was performed without adding the anti-CypB primary antibody. SR, secretory region.

Following the high expression of *CypB* in response to TYLCV in viruliferous midguts, we tested whether this response involves direct interaction between TYLCV and CypB protein. Two methods were used to test possible interaction. First, an IC-PCR assay was used and the controls included TYLCV-infected tomato leave extracts which were applied to microtiter well-plates coated with anti-CypB antibody (**Figure 4**). Other controls included extracts from non-viruliferous whiteflies and from TYLCV-uninfected plants which were applied to plates coated with anti-TYLCV CP antibody (**Figure 4**). The PCR results obtained with these controls were all negative. Extracts from viruliferous whiteflies applied to plates coated with anti-CypB antibody (**Figure 4**), or extracts from TYLCV-infected tomato applied to plates coated with anti-TYLCV CP antibody (**Figure 4**), provided positive PCR results, suggesting a specific interaction between CypB and TYLCV, very likely via the formation of CypB and TYLCV complexes. Second, co-immunolocalization of TYLCV (CP) and CypB using specific polyclonal antibodies, in the insect midguts, salivary glands, and eggs. As seen in **Figure 5**, colocalization of both CypB and TYLCV was observed as yellow patches in viruliferous whitefly midgut epithelial cells (**Figure 5**), and in primary salivary glands and oocytes (**Figure 6**), while the controls demonstrated the specificity of the procedure and the primary antibodies used (**Figure 5**).

### Feeding Whiteflies with Anti-CypB Antibody or with the Cyp Inhibitor Cyclosporin A Decrease TYLCV Transmission

**FIGURE 7:**
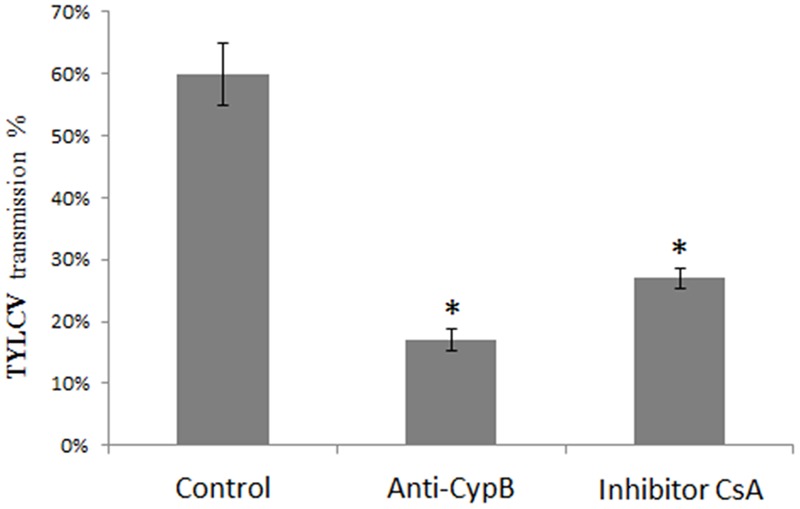
**Decrease in TYLCV transmission rates after feeding of adult whiteflies on artificial medium containing 25% sucrose (control), artificial medium containing anti-CypB antibodies (anti-CypB) and the cyclophilin inhibitor CsA, for 24 h, followed by feeding on the TYT plants for 48 h, before performing transmission experiments of one insect/plant as detailed in the text.** Data shown are the means and standard errors from three independent experiments. Asterisks indicate statistically significant differences with *P* < 0.05 between transmission experiments compared to the control.

Transmission experiments were performed with whiteflies fed with anti-CypB antibody to further investigate the involvement of CypB in TYLCV transmission. *B. tabaci* B adults were first fed with artificial medium containing CypB antibody for 24 h and subsequently fed on TYLCV-infected tomato plants for 24 h. In the control experiments, adult whiteflies were fed with 25% sucrose, followed by feeding on infected plants. In the antibody-feeding experiment, 6 out of 20 plants in the first experiment and 5 out of 20 plants in the second and third experiments became infected, while 13 plants out of 20 in the first and second experiments, and 10 plants out of 20 in the third experiment became infected after feeding with 25% sucrose (control). Altogether, 17% transmission rates, on average, were obtained after feeding with anti-CypB antibody, compared to 60% transmission when the whiteflies were fed with 25% sucrose solution (**Figure 7**).

In another set of transmission experiments, adult *B. tabaci* B whiteflies were fed the Cyp inhibitor CsA to further investigate the involvement of Cyp in TYLCV transmission. The whiteflies were fed with CsA for 24 h, or with 25% sucrose as a control, and the insects from both groups were caged with TYLCV-infected plants for a 48 h acquisition access period. Interestingly the transmission rates dramatically decreased, with 3 out of 20 plants in the first and second experiments and 4 out of 20 plants in the third experiment became infected, when the insects were fed with Cyp inhibitor, and altogether, 27% transmission rates, on average, were obtained, compared to 60% when the whiteflies were fed with 25% sucrose solution (**Figure 7**).

**FIGURE 8:**
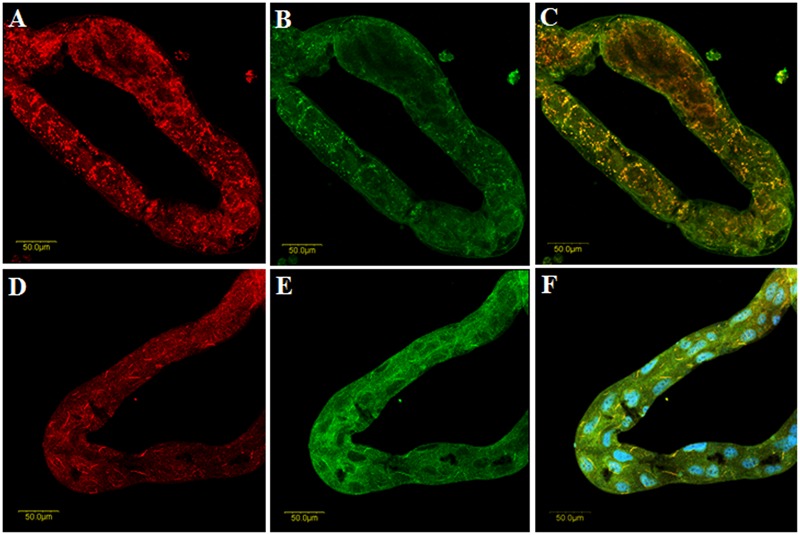
**Colocalization of TYLCV (A**, red) and CypB (**B**, green) in midguts dissected from VBt fed on 25% sucrose. **(C)** Shows the yellow spots where CypB and TYLCV co-localize. **(D)** Shows immunolocalization of TYLCV after feeding with the CypB inhibitor CsA and **(E)** Shows immunolocalization of CypB after feeding with the CsA inhibitor. **(F)** Is an overlay of **(D** and **E)**. Blue is DAPI staining of the nuclei.

The following experiment was conducted for demonstrating the internalization of the anti-CypB antibody by whiteflies and testing the effect of the CypB inhibitor on colocalization of CypB and/or TYLCV and their interaction. Midguts were dissected from whiteflies fed on the first antibody against CypB and the CypB inhibitor CsA, and then were fed goat-anti rabbit Cy2 secondary antibody. Typical CypB-TYLC colocalization was obtained when viruliferous whiteflies were fed only sucrose (**Figures 8A–C**), while atypical signal of TYLCV and no signal of CypB were observed in the midguts dissected from whiteflies fed with the Cyp inhibitor CsA (**Figure 8**).

## Discussion

Successful infection cycle for whitefly-transmitted begomoviruses depends on the efficiency of acquisition, transmission, and retention of the virus in the whitefly vector (Rosen et al., 2015). Although these interactions have been studied for many years, very little is known about the molecular mechanisms that govern the transmission of this group of viruses by their hemipteran vectors. Several insect and endosymbiont proteins have been implicated in the recognition and translocation of TYLCV virions in the vector (Gottlieb et al., 2010; Gotz et al., 2012). TYLCV CP was shown to be the only and essential viral protein involved in virus transmission (Hofer et al., 1997). Two additional proteins were shown to interact with TYLCV CP. First, GroEL protein from the secondary symbiont *Hamiltonella* of *B. tabaci* B, which has been shown to be required for protecting virions from rapid proteolysis in the insect hemolymph (Gottlieb et al., 2010), thus ensuring safe and efficient transmission of the virus. Second, Heat-Shock Protein 70 HSP70 whose expression was shown to be induced upon virus retention and restricts virus transmission (Gotz et al., 2012).

Recently, peptidyl-prolyl isomerases proteins (PPIases or Cyps) have been implicated in B/CYDV circulation and transmission by aphids (Tamborindeguy et al., 2013), and were hypothesized to play a role in chaperoning these viruses to various membrane bound vesicles (Yang et al., 2008). Cyps are important in the proper folding of proteins and as modulators for human virus replication (Frausto et al., 2013). Furthermore, Cyps were shown to interact with the capsid protein of the human immunodeficiency virus type 1 (HIV-1; Schaller et al., 2011) and influenza A virus M1 protein (Liu et al., 2009) and were shown to play a key role in the viral replication cycle. In this study, we investigated whether Cyps from *B. tabaci* B have a role in begomovirus transmission. We used molecular, biological and microscopy approaches to show that *CypB* gene expression is altered upon TYLCV acquisition and retention by *B. tabaci*, and close proximity and possible physical interaction with the virus were verified. First, we have analyzed the expression of various Cyp genes in viruliferous and non-viruliferous whiteflies using a qRT-PCR approach (**Figures 3A–C**). Interestingly, the three *Cyp* genes tested in this study, *CypB, Cyp*D, and *Cyp*G, showed generally down regulation in their expression in R+ viruliferous whole insects, however, their expression was not similar and fluctuated in the R- strain. When the expression of Cyps was measured in midguts, only the expression of *CypB* was significantly induced by twofold in viruliferous R+ whiteflies, compared to the uninfected insects. Our results also show that infection with *Rickettsia* results in lower expression of *Cyp* genes after TYLCV acquisition. These results indicate that the role of Cyp proteins might involve multitrophic interactions between insect proteins, endosymbionts, and the virus CP, especially that when the expression of the three *Cyp* genes was measured in R+ and R- insects, without TYLCV acquisition, their expression was always significantly higher in the R+ insects (**Figures 3A–C**). These results support a previous raised hypothesis which suggested that *Rickettsia* plays an important role in TYLCV-*B. tabaci* interactions (Kliot et al., 2014). Further research will be required to verify the role of *Rickettsia* in altering expression of *Cyp* genes before and post TYLCV acquisition.

The expression results of *CypB* in the midgut were different from whole body results (**Figure 3D**), and combined with previous observations that the majority of TYLCV particles are translocated from the filter chamber in the midgut to the hemolymph (Ghanim et al., 2009; Skaljac and Ghanim, 2010; Gotz et al., 2012), suggest that Cyp might play an important role in TYLCV-*B. tabaci* interactions in the gut. Such roles include transport and translocation to the hemolymph. If indeed this is the role of Cyp, it was hypothesized that direct interaction between CypB and TYLCV CP might occur in the insect, specifically in the gut and the salivary glands, major sites along the transmission pathway. To demonstrate such interaction, we performed IC-PCR (**Figure 4**) and co-immunostaining (**Figure 5**). Both methods clearly demonstrated that CypB and TYLCV are likely to interact *in vivo*. The colocalization of CypB and TYLCV in the filter chamber, ceacae and the midgut further support this hypothesis and show close proximity of the proteins in these organs. The filter chamber is likely be the site where most of these interactions with the virus occur, suggesting that Cyp might play an important role in translocating the virus to the hemolymph. These colocalization results and the possible shuttling of the virus by Cyp in the filter chamber is supported by the increase in CypB concentration when measured by qRT-PCR in the midgut. Further subcellular localization experiments may confirm these colocalization results and the role of CypB protein in TYLCV-*B. tabaci* interactions.

Our results further confirmed CypB colocalization with TYLCV in the primary salivary glands of viruliferous insects (**Figure 6**). Gottlieb et al. (2010) demonstrated that *Hamiltonella*-GroEL protein plays a crucial role in safeguarding the virus in the hostile environment of the hemolymph, while proteins that aid in virus translocation from the midgut to the hemolymph and from the hemolymph to the salivary glands have not been described. CypB protein is a candidate that could have a major role in these two organs with regard to TYLCV transmission. Our results also show that CypB-TYLCV colocalization was observed in eggs from viruliferous whiteflies (**Figure 6**). A previous study has suggested that TYLCV is transovarially transmitted (Ghanim et al., 1998) through the reproductive system, and the interaction with CypB can be an important factor that determines the efficiency of TYLCV transmission from one generation to another through the reproductive system.

To further test whether the observed interaction are instrumental in influencing the transmission of the virus to test plants, we conducted virus transmission experiments after feeding adult whiteflies with anti-CypB antibodies and the CypB inhibitor CsA. The results showed that TYLCV transmission was reduced by 43% and 33 after feeding with anti-CypB and CsA, respectively (**Figure 7**). Those results are supported by the colocalization results in the midgut, in which decrease in the virus and CypB levels was observed, and their shape turned abnormal when fed with CsA. CsA is a general inhibitor of cyclophilins and it is possible that other cyclophilins in the insect were also affected following the feeding. However, since our results from other experiments indicate that CypB is possibly the protein involved in *B. tabaci-*TYLCV interactions, it is likely that the effect of CsA of virus transmission and localization is related to CypB. In aphids, two CypB proteins, S28 and S29, were identified in populations of *Schizaphis graminum* and those populations showed differences in their ability to transmit CYDV-RPV, and were related to the circulative transmission of luteoviruses, suggesting that those proteins play similar roles in whiteflies and aphids. The reduction in the virus and CypB levels, and their abnormal shape after feeding with the inhibitor suggests that CypB acts also to stabilize the virus in the gut, and might be aiding the virus to avoid or combat the insect immune responses. Recently, it was demonstrated through structural analyses that CycA stabilizes the HIV-1 capsid and is recruited to facilitate HIV-1 infection (Liu et al., 2016), suggesting that CypB might have a similar role with TYLCV in the whitefly.

## Conclusion

We demonstrated in the present study an important role of *B. tabaci* B CypB protein in TYLCV circulative transmission. The results further suggest that this interaction might not only be relevant to the virus transmission to plants but also for the virus transovarial transmission between generations. Further studies in this system will focus on functional analysis of the role of CypB in the interaction with TYLCV including *CypB* gene silencing in whiteflies, the exact role of the protein in the virus translocation in the different insect organs, and whether this CypB-TYLCV interaction is important for mediating the virus interaction with other insect proteins during the transmission process.

## Author Contributions

Conceived and designed the experiments: MG and SK. Performed the experiments: SK and MG. Analyzed the data: SK and MG. Contributed materials/analysis tools: MG. Wrote the paper: SK and MG.

## Conflict of Interest Statement

The authors declare that the research was conducted in the absence of any commercial or financial relationships that could be construed as a potential conflict of interest.

## Footnotes

1 http://www.ictvonline.org/virustaxonomy.asp

2 http://www.ncbi.nlm.nih.gov/blast

3 http://www.expasy.org/

4 http://swissmodel.expasy.org/

5 http://www.mbio.ncsu.edu/BioEdit
